# Supplementary material for: Role and mechanism of NCAPD3 in promoting malignant behaviors in gastric cancer
Source: Front Pharmacol. 2024 Apr 22;15:1341039. doi: 10.3389/fphar.2024.1341039 (PMC11070777; doi:10.3389/fphar.2024.1341039)
Supplement: Supplementary file 11 [file DataSheet2.ZIP › GSEA/Transcipt factor/my_analysis.Gsea.1599461814734/CEBP_Q3.html]

Details for gene set CEBP\_Q3[GSEA]

|  || Dataset | filtered\_dataset.sample\_info.cls#WT\_versus\_NCAPD3\_MUT |
| Phenotype | sample\_info.cls#WT\_versus\_NCAPD3\_MUT |
| Upregulated in class | NCAPD3\_MUT |
| GeneSet | CEBP\_Q3 |
| Enrichment Score (ES) | -0.42537695 |
| Normalized Enrichment Score (NES) | -1.946587 |
| Nominal p-value | 0.0025125628 |
| FDR q-value | 0.080581024 |
| FWER p-Value | 0.717 |
Table: GSEA Results Summary

  

Fig 1: Enrichment plot: CEBP\_Q3      
 Profile of the Running ES Score & Positions of GeneSet Members on the Rank Ordered List

  

| SYMBOL | TITLE | RANK IN GENE LIST | RANK METRIC SCORE | RUNNING ES | CORE ENRICHMENT || 1 | 5728 | PTEN | 285 | 0.606 | -0.1467 | No |
| 2 | 4281 | MID1 | 294 | 0.595 | -0.0954 | No |
| 3 | 8125 | ANP32A | 326 | 0.578 | -0.0622 | No |
| 4 | 2997 | GYS1 | 376 | 0.548 | -0.0449 | No |
| 5 | 10492 | SYNCRIP | 779 | 0.334 | -0.3016 | No |
| 6 | 10856 | RUVBL2 | 847 | 0.257 | -0.3252 | No |
| 7 | 9388 | LIPG | 937 | -0.361 | -0.3545 | No |
| 8 | 7067 | THRA | 976 | -0.392 | -0.3442 | No |
| 9 | 1604 | CD55 | 1090 | -0.468 | -0.3805 | Yes |
| 10 | 8444 | DYRK3 | 1131 | -0.502 | -0.3612 | Yes |
| 11 | 604 | BCL6 | 1136 | -0.504 | -0.3157 | Yes |
| 12 | 2119 | ETV5 | 1176 | -0.545 | -0.2914 | Yes |
| 13 | 8535 | CBX4 | 1184 | -0.555 | -0.2433 | Yes |
| 14 | 51776 | ZAK | 1196 | -0.571 | -0.1964 | Yes |
| 15 | 8863 | PER3 | 1227 | -0.601 | -0.1602 | Yes |
| 16 | 26137 | ZBTB20 | 1244 | -0.622 | -0.1121 | Yes |
| 17 | 1649 | DDIT3 | 1341 | -0.779 | -0.1063 | Yes |
| 18 | 51703 | ACSL5 | 1353 | -0.800 | -0.0375 | Yes |
| 19 | 4622 | MYH4 | 1355 | -0.811 | 0.0395 | Yes |
Table: GSEA details [plain text format]

  

Fig 2: CEBP\_Q3      
 Blue-Pink O' Gram in the Space of the Analyzed GeneSet

  

Fig 3: CEBP\_Q3: Random ES distribution      
 Gene set null distribution of ES for **CEBP\_Q3**
